# Association between subjective orthodontic treatment need, oral health-related quality of life, and occlusal deviations in a cohort of 17-year-old adolescents in Finland

**DOI:** 10.2340/aos.v84.43565

**Published:** 2025-05-13

**Authors:** Heidi Arponen, Eija Latvakoski, Linnea Närhi, Anna-Sofia Silvola

**Affiliations:** a Department of Oral and Maxillofacial Diseases, University of Helsinki, Helsinki, Finland; b Head and Neck Center, Helsinki University Hospital, Helsinki, Finland; c Wellbeing Services County of Vantaa and Kerava, Vantaa, Finland; d Wellbeing Services County of North Ostrobothnia, Pohde, Finland; e Research Unit of Population Health, University of Oulu, Finland; f Medical Research Center (MRC) Oulu, Oulu, Finland; g Oulu University Hospital, Oulu, Finland

**Keywords:** Oral health-related quality of life, orthodontics, malocclusion, satisfaction, OHIP-14

## Abstract

**Objective:**

To investigate the relationship between self-perceived orthodontic treatment need, satisfaction with dental esthetics and occlusion, oral health-related quality of life (OHRQoL), occlusal deviations, and orthodontic treatment history of 17-year-olds.

**Material and methods:**

Clinical examination and standardized questionnaire were completed by 108 adolescents from two municipal clinics. OHRQoL was measured with the Oral Health Impact Profile (OHIP-14) and malocclusion severity using the Peer Assessment Rating (PAR) Index. Associations between the variables were analyzed with Spearman’s rank correlation and linear regression.

**Results:**

Of the participants, 55% had either undergone orthodontic treatment or were in treatment. The level of self-perceived treatment need was low and satisfaction with esthetics and functionality of occlusion high. Painful aching in the mouth was the most reported OHIP item, followed by self-consciousness. Subjective treatment need and PAR correlated positively (*r*_s_(95) = 0.389, *p* < 0.001), whereas the OHIP score and PAR score showed no association. Subjective treatment need, satisfaction, or treatment history did not predict the OHIP score.

**Conclusions:**

Majority of the adolescents were satisfied with their dental esthetics and occlusal function irrespective of past orthodontic treatment history. OHRQoL was poorer in adolescents with subjective orthodontic treatment need. Those with the greatest subjective treatment need had the most severe malocclusion.

## Introduction

In Nordic countries, orthodontic treatment of children and adolescents is largely publicly funded. Minor deviations from normal occlusion and esthetic concerns do not usually fulfill the criteria for publicly funded care. However, defining malocclusion and orthodontic treatment need is not unequivocal. In Finland, all dental care, including orthodontics, is available for children and adolescents under the age of 18 years free of charge. Publicly funded orthodontic care is offered universally to individuals with a severe malocclusion, as evaluated by a modified Treatment Priority Index [1, 2]. Nevertheless, because of limited and varying resources, some differences have been observed in the orthodontic practices of different municipalities [3]. The treatment need evaluation emphasizes functional impairments, as the index used does not have an esthetic component. Similarly, the primary aim of the treatment is improving occlusal function. In general, patients tend to expect orthodontic treatment to improve their dental esthetics and social attractiveness [4, 5]. Thus, the goals set by the orthodontists working in public health care might be in contrast to patient expectations [4, 5].

Oral health-related quality of life (OHRQoL) is a multidimensional concept based on the World Health Organization definition that considers health as the state of complete physical, mental, and social well-being [6]. OHRQoL considers self-perceived influence of oral diseases and dental interventions [7]. Although OHRQoL does not reveal clinical oral status as such, it allows for identification of an individual’s perception of oral health and the impact of dental treatment on the individual’s life [8]. To measure OHRQoL, different indices have been developed [9]. The short-form of the Oral Health Impact Profile (OHIP-14) is a widely used general OHRQoL measure and is validated in many languages [8, 10, 11]. Individuals having esthetic or functional concerns related to malocclusion report higher OHIP scores than those satisfied with their occlusion [12]. Previous research indicates an association between occlusal deviations and OHRQoL and affirms the positive impact of orthodontic intervention [13–15]. However, providing orthodontic treatment free of charge to everyone based solely on subjective need would scarcely be feasible. Thus, it is essential to assess the attainable improvements in occlusion with existing resources of publicly funded orthodontic care, as well as the correlation between received orthodontic treatment and OHRQoL.

This observational multicenter study aimed to evaluate subjective orthodontic treatment need, satisfaction with dental esthetics and occlusal function, as well as OHRQoL in relation to occlusal deviations, and the orthodontic treatment history of adolescents in two Finnish municipalities. The study hypothesis was that adolescents with high subjective treatment need have low satisfaction with dental esthetics and occlusal function, as well as low OHRQoL and deviation from ideal occlusion.

## Materials and methods

The data collection was conducted in two Finnish cities: one located in the capital area (Vantaa) and one in northern Finland (Oulu). Vantaa is Finland’s fourth largest city by population and Oulu the fifth. In both cities, an experienced orthodontist carried out the examinations. Prior to the start of the study, the orthodontists performing the clinical examinations (H.A. and E.L.) were trained and calibrated to determine inter-examiner reliability. The regional medical research ethics committee of the Wellbeing Services County of North Ostrobothnia had previously given ethical consent (EETTMK: 47 /2021), and the Wellbeing Services County of North Ostrobothnia and the Wellbeing Services County of Vantaa and Kerava had granted research permission (3/2022). Informed consent was obtained from all participants.

### Study sample

**Figure 1:**
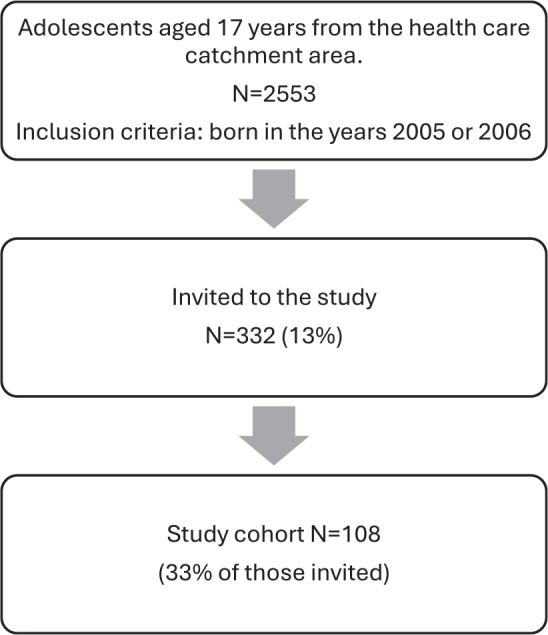
Study population flow chart.

Of the adolescents, born in the years 2005 or 2006 and residing within the health-care center’s catchment area of Myyrmäki, Vantaa (*N* = 559), and Oulu (*N* = 1,994), 332 were randomly invited to participate in a clinical study conducted between April 2022 and February 2024 (Figure 1). The age group was considered ideal for assessing subjective satisfaction as, by late adolescence, most individuals have completed their dental development and craniofacial growth and are mature enough to provide informed opinions about their orthodontic treatment need, dental appearance, and occlusal functionality. Data collection included a standardized questionnaire, clinical dental and oral examination, and intraoral scanning. In addition, data on current or previous orthodontic treatment were collected from the patient records. The treatment need of those with orthodontic treatment history had been evaluated previously with the Treatment Priority Index.

### Questionnaire

All participants completed a questionnaire comprising background information, a report on previous or current orthodontic treatment, an assessment of subjective need for orthodontic treatment, satisfaction with dental esthetics and occlusal function, and the OHIP-14 questionnaire measuring OHRQoL. Background information included current study place of the participant (vocational education/high school/working/other) and the highest level of education of their parents (elementary school/high school or vocational education/high school followed by vocational education/polytechnic or university degree).

The respondents were asked to assess a self-perceived need for orthodontic treatment with a numeric 10-grade scale from 0 (no treatment need) to 10 (very high treatment need). Satisfaction with dental esthetics and occlusal function was asked with the additional questions: ‘How satisfied are you with your dental esthetics?’ and ‘How satisfied are you with your occlusal function?’ and rated in a similar manner on a scale from 0 = very dissatisfied to 10 = very satisfied.

The OHIP-14 questionnaire, which covers dimensions of functional limitation, physical pain, psychological discomfort, physical disability, psychological disability, social disability, and handicap, has been found to be valid and reliable in its Finnish version [8, 16]. Each question was answered on a five-point Likert scale (0 = never, 1 = hardly ever, 2 = occasionally, 3 = fairly often, 4 = very often), with an optional ‘I don’t know’. The OHIP-14 severity score (potential range 0–56) was calculated by summing the ordinal values for all the 14 items. A high OHIP-14 severity score indicates low OHRQoL.

### Clinical examination

The clinical examination included registration of occlusal deviations and function of occlusion. Sagittal molar and canine relationships were classified as either Angle Class I, Class II, or Class III for both sides separately. Overjet and overbite were measured in millimeters and, in addition, the lower incisor coverage by the upper incisors was defined as either under or over two-thirds of the lower incisor crown height. Traumatic deep bite was recorded as present or absent. Crossbite and scissors bite were registered for each tooth and dichotomized as present/absent. Upper and lower midline shift was recorded as under, or equal to or more than 3 mm from facial midline. Occlusal shift was measured with an accuracy of half a millimeter. Laterotrusion was recorded as following: 1 = canine guidance, 2 = group guidance, and 3 = other, including balance side contacts. Protrusion was recorded as 1 = anterior guidance, 2 = anterior guidance with posterior contact or 3 = only posterior contacts.

### Malocclusion severity

Digital scans (*n* = 102) or a plaster cast model (*n* = 6) were obtained for each participant. Digital scans were acquired using Planmeca Emerald (Planmeca Oy, Helsinki, Finland) and analyzed with Romexis^Ò^ Model Analyser software (Planmeca Oy, Helsinki, Finland). Of the intraoral scans, 11 were excluded because of poor quality and one scan had not been saved in the patient files. Previous literature has verified a good agreement between manual and digital methods in inter-arch occlusal measurements [17]. The models were analyzed using the Peer Assessment Rating (PAR) index by a single examiner (H.A.) trained by a formally calibrated orthodontist (A.-S.S.) [18, 19]. The PAR index has been developed to assess malocclusion severity and to evaluate orthodontic treatment outcome and any possible residual treatment need [15, 20]. PAR is a cumulative index considering all deviations from normal occlusion. The index consists of five components: anterior segment, buccal occlusion, overbite, overjet, and centerline. The component scores were weighted according to British weighting [18]. The PAR total score is a summary score representing all occlusal deviations, with a higher score indicating a more severe deviation from an ideal occlusion [19].

Intra-examiner reliability of the PAR measurements was determined by duplicating scoring of 20 models at a minimum interval of 2-weeks. The intra-class correlation coefficient (ICC) two-way mixed-effects model was used to evaluate the reliability among the repeated measurements, each model independently analyzed. Cases with missing data due to poor quality of intraoral scans were excluded from analysis and only complete cases were considered.

### Statistical analysis

For the reliability analysis, ICC estimates and their 95% confidence intervals (CIs) were calculated based on a mean-rating (κ = 2), absolute-agreement, 2-way mixed-effects model. Cohen’s kappa value analysis was run to determine whether there was agreement between the two orthodontists clinically examining the study participants.

Differences in subjective measures and normatively measured occlusal deviations in individuals with and without orthodontic treatment history, as well as those awaiting orthodontic treatment, were assessed with a Mann-Whitney *U* test using the SPSS software (IBM, version 27, Chicago, IL, USA). Associations between the variables were analyzed with Spearman’s rank correlation analysis, missing cases excluded pairwise. The effect of subjective treatment need, satisfaction with dental esthetics and occlusal function, overjet, overbite, PAR total score, and previous orthodontic treatment history (independent variables) on OHIP-14 (dependent variable), were further analyzed with a simple linear regression analysis. The assumption of normality of residuals was evaluated using the Kolmogorov-Smirnov test. Potential outliers, defined as values greater than +3 or less than -3, were identified using scatterplots of residuals, and one outlier was removed from the linear regression model.

Missing data in the background questionnaire were missing at random and thus a pairwise deletion was applied, and all the available data were included in the final analysis. When a respondent had answered ‘I don’t know’ to a single OHIP-question, the question group median was used as a value. One response sheet was excluded from the analysis due to ‘I don’t know’ answer to all questions (available-case analysis).

### Sample size calculation

Sample size calculation was based on the national average of 28% of all 12-year-olds receiving orthodontic care in the year 2022, as recorded by the Finnish Institute for Health and Welfare [21]. Data collection aimed for a sample size of 126 which, with an 80% confidence level and 5% margin of error, would be representative of a population of 2 553 adolescents aged 17 years living in the chosen cities.

## Results

The final study group comprised 108 adolescents (47 males) aged between 16.5 and 18.0 years meeting the inclusion criteria and consenting to participate. They represented 4.2% of eligible adolescents (Figure 1, Table 1). Post hoc power analysis comparing the sample with the eligible population size revealed a margin of error of ± 2.4 for an 80% confidence level.

**Table 1:** Study population characteristics and orthodontic treatment history (*N* = 108).

	*N* (%)	Mean	Range
Males	47 (44)		
Mean age/years		17.4	16.7–18.0
In orthodontic treatment	10 (9)		
Orthodontic treatment planned	7 (6)		
Previous orthodontic treatment	59 (55)		
Treatment duration/years		3.2	0.3–8.3
Mean age at onset of treatment /years		10.7	5.9–17.1
Orthodontic appliance type (*N* = 59)			
Fixed appliances	34 (58)		
Maxillary expansion (QH/RME)	6 (10)		
Headgear/facemask	19 (32)		
Functional appliance	13 (22)		
Eruption guidance appliance	8 (14)		
Space maintenance arch	18 (31)		
Study place (*N* = 106)			
Vocational school	35 (33)		
High school	67 (63)		
Working	2 (2)		
Other	2 (2)		
Mother’s highest education (*N* = 95)			
Elementary school	2 (2)		
High school or vocational education	28 (29)		
High school followed by vocational education	16 (17)		
University or polytechnic	49 (52)		
Father’s highest education (*N* = 94)			
Elementary school	3 (3)		
High school or vocational education	28 (30)		
High school followed by vocational education	11 (12)		
University or polytechnic	52 (55)		

### Inter- and intra-examiner reliability

There was a substantial strength of agreement between the two orthodontists’ judgements in assessing the Angle classification (κ = 0.776, 95%CI [0.366, 1.186], *p* < 0.001). Good reliability was found in overjet measurements (ICC = 0.865 [df(19) 95%CI 0.659, 0.947], *p* < 0.001) and excellent reliability in overbite measures (ICC = 0.945 [df(19) 95%CI 0.860, 0.978], *p* < 0.001). Perfect agreement was reached in determining traumatic deep bite crossbite, and scissors bite, and midline deviation (κ = 1.00, 100% agreement). Lateral guidance registration agreement was low between the examiners (κ = 0.221 95%CI [-0.165, 0.607]), whereas the protrusion guidance registration agreement was good (κ = 0.828 [95%CI 0.503, 1.15]).

A high degree of reliability was found between the repeated PAR measurements. The average measure ICC was 0.938 with a 95% CI (0.848, 0.975 (df(20) = 16.189, *p* < 0.001).

### Subjective treatment need, satisfaction, and oral-health-related quality of life

**Figure 2:**
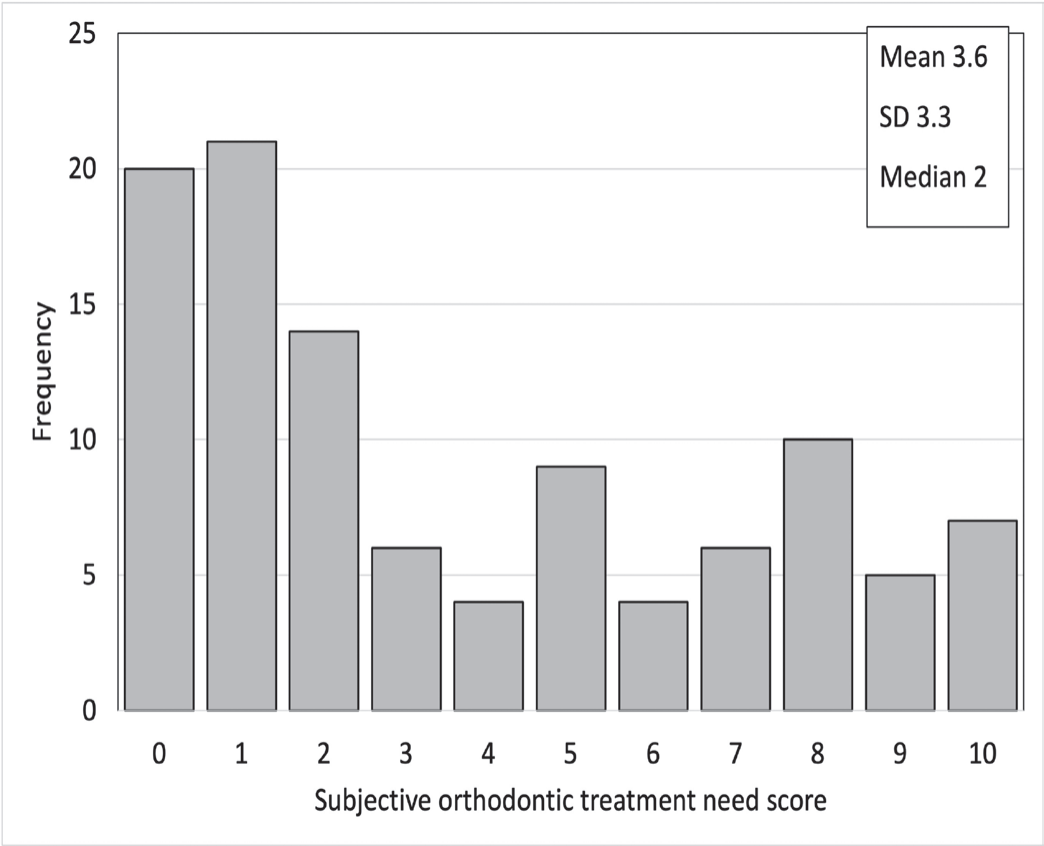
Frequency distribution of subjective orthodontic treatment need evaluation on a scale from 0 = no treatment need to 10 = very high treatment need (N = 106).

**Figure 3:**
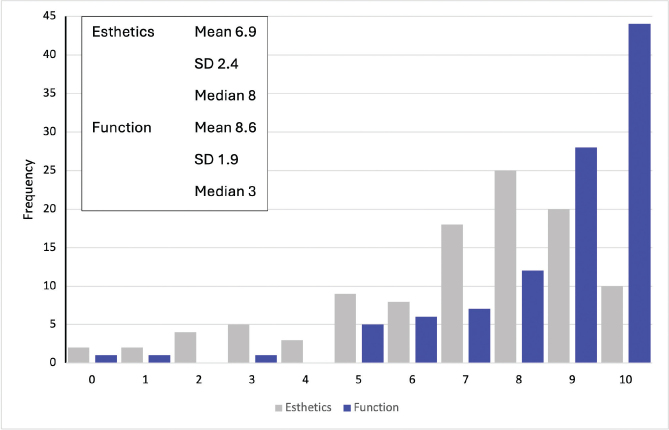
Satisfaction with dental esthetics and occlusal function evaluated with a 10-grade scale from 0 = very dissatisfied to 10 = very satisfied (N = 106 and N = 105 respectively).

Figures 2 and 3 display the distributions of subjective orthodontic treatment need and satisfaction with dental esthetics and occlusal function, respectively. The average subjective treatment need was 4 out of 10, on a scale where 10 represented very high treatment need. The average satisfaction with dental esthetics was 7 out of 10, and satisfaction with occlusal function was 9 out of 10, on a scale where 10 represented very satisfied.

The average subjective treatment need was 8 out of 10 (*N* = 10) among participants undergoing orthodontic treatment and 5 out of 10 among those awaiting treatment (*N* = 7).

Among the participants undergoing orthodontic treatment, the average satisfaction with dental esthetics was similar to the whole cohort average (7 out of 10), whereas satisfaction with occlusal function was lower (8 out of 10). Among participants awaiting orthodontic treatment, the average satisfaction with dental esthetics was 5 out of 10, and with occlusal function 6 out of 10. The distribution of subjective orthodontic treatment need and satisfaction with dental esthetics and occlusal function grades was not statistically different between those under orthodontic treatment and those with finished treatment (*U* = 1465.0, *p* > 0.387, *U* = 1533.5, *p* = 0.187, and *U* = 1350.5, *p* = 0.753 respectively).

**Figure 4:**
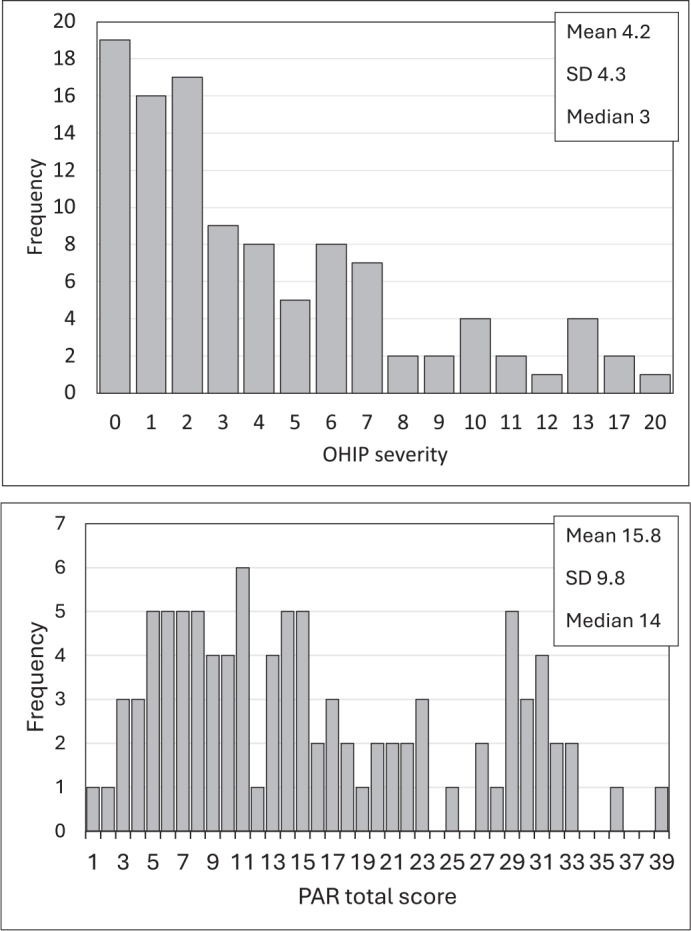
Distributions of the Oral Health Impact Profile (OHIP-14) scores (N = 107) and the total Peer Assessment Rating (PAR) scores (N = 96).

**Figure 5:**
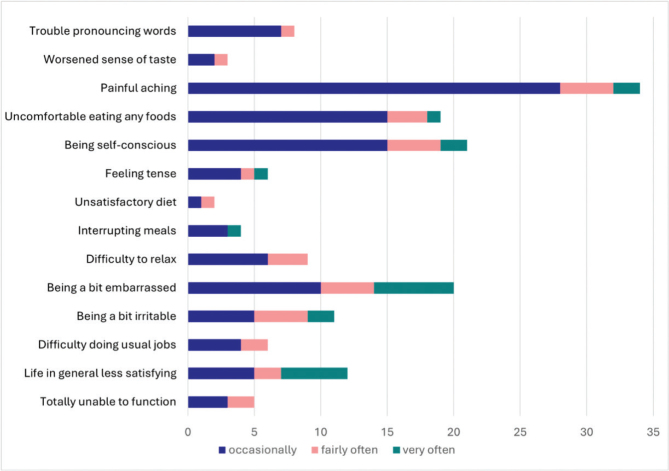
Mean score values for Oral Health Impact Profile questions (1 to 14) of 107 individuals (one response sheet was excluded due to missing values).

Mean value of OHIP severity was 4.2 (range 0–20) (Figure 4). Painful aching in the mouth was the most reported OHRQoL impact, followed by self-consciousness (Figure 5).

### Occlusal deviations

The majority of the participants (77%) had a Class I molar relationship (Table 2). Crossbite was the most common malocclusion followed by a Class II relationship and deep bite. Figure 4 presents the observed variation in PAR total score. The mean PAR total score of the adolescents in Oulu was significantly lower compared with that of Vantaa (mean score 10.8 and 17.5 respectively, *p* = 0.007).

**Table 2:** Prevalence of different malocclusions and occlusal traits in the studied sample (N = 108).

Occlusal trait	*N* (%)
Angle class I	83 (77)
Angle class II	21 (19)
Angle class III	5 (4.6)
Asymmetric molar relationship	31 (29)
Canine relationship class I	84 (78)
Canine relationship class II	19 (18)
Canine relationship class III	5 (4.6)
Increased overjet (> 5 mm)	12 (11)
Negative overjet (< 0 mm)	2 (1.9)
Anterior open bite	3 (2.8)
Deep bite (lower incisor coverage > 2/3 of the crown height)	15 (14)
Traumatic deep bite	9 (8.3)
Crossbite	16 (15)
Scissor bite	8 (7.4)
Upper midline deviation more than 3mm from facial midline	3 (2.8)
Laterotrusion; other than canine guidance or group function in either side	11 (10)
Protrusion; other than anterior group guidance	20 (19)
Functional shift	12 (11)

### Orthodontic treatment history

Of the participants, 55% had either undergone previous orthodontic treatment or were in treatment according to patient records and/or questionnaire (Table 1). Regional differences were present in orthodontic treatment history: the prevalence of orthodontic treatment history was 45% in Vantaa and 77% in Oulu (*r*_s_(106) = 0.282, *p* = 0.003). Detailed orthodontic treatment data were available on 80% of those with treatment history. The average age at the start of treatment was 10.7 years (11.1 years in Vantaa and 10.0 years in Oulu) and the average treatment time was 3.2 years. Nine individuals had interrupted orthodontic treatment. Girls had received treatment as equally often as boys (*p* = 0.423). All the participants in treatment or awaiting treatment were living in Vantaa. Two participants were scheduled for orthognathic surgery, and two for prosthodontic treatment because of oligodontia. One of the participants awaiting orthodontic treatment had received treatment previously.

### Association between the variables

Adolescents with high subjective treatment need were less satisfied with their dental esthetics (*r*_s_(106) = -0.518, *p* < 0.001) and occlusal function (*r*_s_(104) = -0.416, *p* < 0.001) and reported higher OHIP severity scores (*r*_s_(105) = 0.335, *p* < 0.001). The OHIP severity score correlated inversely with subjective satisfaction with dental esthetics and occlusal function (*r*_s_(106) = -0.408, *p* < 0.001 and *r*_s_(105) = -0.494, *p* < 0.001 respectively).

High subjective treatment need correlated positively with large overjet (*r*_s_(106) = 0.249, *p* = 0.010) and high PAR total score (*r*_s_(95) = 0.433, *p* < 0.001). The OHIP severity and PAR total score did not show correlation (*p* = 0.337). However, individuals who reported being embarrassed, been irritable, and feeling that life in general is less satisfying because of problems with teeth or mouth had a high PAR total score (*r*_s_(95) = 0.340, *p* = 0.001, *r*_s_(92) = 0.228, *p* = 0.029, and *r*_s_(93) = 0.322, *p* = 0.002 respectively). Large overjet and overbite correlated positively (*r*_s_(108) = 0.404, *p* < 0.001).

No correlation was found between subjective treatment need and orthodontic treatment history (*r*_s_(104) = 0.057, *p* = 0.568, *U* = 1465, *p* = 0.387). Similarly, no association was found between the OHIP severity score and orthodontic treatment history (*p* = 0.568). The seven individuals awaiting orthodontic treatment reported significantly lower subjective satisfaction with occlusal function than those with either treatment completed or no treatment need (*U* = 45.00, *p* = 0.006). Satisfaction with dental esthetics and subjective treatment need or OHIP severity did not differ between those awaiting treatment and those with either treatment completed or no treatment need (*p* > 0.05).

After removing the extreme outlier from the linear regression, an assumption check indicated that residuals were approximately normally distributed and homoscedastic. The overall regression was statistically significant (*R*^2^ = 0.204, F(8,80) = 2.560, *p* = 0.015). We found that subjective treatment need, satisfaction with dental esthetics, satisfaction with occlusal function, overjet, overbite, PAR score, or orthodontic treatment history did not predict a high OHIP severity score (*p* > 0.05) (Table 3).

**Table 3:** Simple linear regression coefficients table to assess the relationship between OHIP severity score (dependent variable) and orthodontic treatment history, subjective treatment need, satisfaction with dental esthetics, satisfaction with occlusal function, PAR score, overjet, or overbite (independent variables).

	B	*P*	95% CI
Orthodontic treatment history	1.311	0.119	-0.342, 2.964
Subjective treatment need	0.141	0.397	-0.189, 0.470
Satisfaction with dental esthetics	-0.277	0.231	-0.733, 0.179
Satisfaction with occlusal function	-0.483	0.083	-1.032, 0.065
Overjet	-0.161	0.568	-0.718, 0.397
Overbite	-0.057	0.812	-0.534, 0.420
PAR score	-0.022	0.662	-0.124, 0.079

PAR: Peer Assessment Rating; CI: confidence intervals.

A statistically significant correlation was detected between subjective treatment need and male gender (*r*_s_(105) = 0.246, *p* = 0.011). The educational background of the participants or their parents did not correlate with subjective treatment need or occlusal deviations (*p* > 0.05). However, the current study place of the participants correlated negatively with the orthodontic treatment history (*r*_s_(99) = -0.231, *p* = 0.022), suggesting that adolescents with lower education had received orthodontic treatment more often than those with higher education.

## Discussion

In a publicly funded orthodontic clinic, resources are limited. Given the high demand for treatment, these resources must be directed to those most in need. The aim of the treatment in public health care in Finland is to achieve functionally and esthetically acceptable results, and to eliminate malocclusions that could potentially damage long-term oral health. However, it has not been studied whether these treatment criteria align with patient expectations.

In this study, among the 17- to 18-year-old adolescents, subjective orthodontic treatment need was generally low. The study subjects were generally satisfied with both dental esthetics and, even more so, with their occlusal function. In our cohort, 76% of participants rated their satisfaction with dental esthetics as 5 or higher, indicating levels above the midpoint of the scale, while 92% expressed above-average level of satisfaction with occlusal function. The experienced OHRQoL impacts among adolescents were relatively low. However, there was notable individual variation in the subjective evaluation of satisfaction and OHRQoL.

A previous study, conducted 24 years ago among 18 to 19-year-olds in Vantaa, Finland, reported that 89% of the young adults were very or quite satisfied with their dental esthetics [22]. A high prevalence of satisfaction with occlusal function (91–93%) has previously been observed also among 15–18-year-old adolescents in Finland [23, 24]. In a previous Finnish study, a definite need for treatment, as evaluated by the Index of Orthodontic Treatment Need (IOTN), was found in 15% of the subjects with no difference in IOTN scores between the treated and untreated subjects [22]. In the present study, conducted in the same city, a smaller percentage of adolescents (9%) with a definite treatment need was found, when evaluated using the Treatment Priority Index and the national 10-point-scale criteria.

Individuals’ subjective feeling of well-being has been noted to increase their OHRQoL, whereas certain personality traits are associated with high subjective need of orthodontic treatment [25–27]. Environmental and socioeconomic factors as well as psychological developmental stage might affect OHRQoL in children [28, 29]. Previous findings on the effects of malocclusion on OHRQoL are contradicting [30]. OHRQoL has been suggested to be deficient in children with malocclusion and subjective orthodontic treatment need [31, 32]. Interestingly, however, OHRQoL shows little correlation with orthodontic treatment need in adults [33], and orthodontic treatment does not seem to affect psychological well-being and self-esteem [34]. In our study, those adolescents, who expressed subjective need for orthodontic treatment and dissatisfaction with the dental esthetics and occlusal function, also reported a high incidence of limitations and discomfort caused by oral diseases, as evaluated with OHIP-14. Thus, these findings substantiate the hypothesis put forth in this research.

Previous studies have demonstrated that OHRQoL is higher in adolescents and adults after receiving treatment for malocclusion and in individuals without malocclusion compared to those with a malocclusion [35, 36]. Our results differ from these findings. Our findings imply that the OHRQoL, as measured by OHIP-14, among adolescents is not related to occlusal features but rather is an independent subjective experience.

The large variation in PAR scores observed in our study can be attributed to the diverse orthodontic treatment status of the study participants. Of the participants, 55% had undergone orthodontic treatment. Some of the participants had malocclusion that did not meet the public health care treatment criteria, some had completed their treatment, while others were either currently undergoing treatment or had treatment planned. The PAR scores were significantly higher in Vantaa compared with Oulu, primarily because many participants in Vantaa had not yet received orthodontic treatment. In Vantaa, 22% of adolescents were either in treatment or waiting for orthodontic treatment to begin.

Treated adolescents have been reported to be significantly more often satisfied with their dental esthetics than those who have not received treatment [23]. However, findings on the relationship between satisfaction with occlusal function and treatment history are inconsistent [23, 24]. In the present study, previous orthodontic treatment was neither associated with satisfaction with dental esthetics or occlusal function nor with subjective treatment need. This unexpected finding may be indicative of the nationally implemented selection criteria, whereby orthodontic treatment is provided for functional displacements and esthetic concerns are not prioritized. Compared with the previous study, in which the overall percentage of those having received orthodontic treatment by 18 years of age was 46% [22], our finding regarding treatment prevalence is consistent in Vantaa, but notably larger in Oulu. Other Finnish investigations, conducted among 15–18-year-old adolescents, have found a 27–85% range in prevalence of orthodontic treatment history [3, 23]. Our current findings confirm that significant variation in the extent of orthodontic services provided persists among health centers in Finland. Inhabitants may face inequality if the extent of orthodontic services varies across different municipalities. The higher percentage of treated adolescents in Oulu may be attributed to the greater number of specialist orthodontists in the region compared with Vantaa [37].

A major weakness of our investigation is the limited sample size. The participant rate was low, possibly resulting in self-selection bias, as those more focused on their teeth and occlusion and those familiar with orthodontic care, were more likely to participate. The suspected bias in our sample size was reflected in a higher than previously recorded national average of adolescents with orthodontic treatment history. However, this limitation would have affected the outcome measures only limitedly, as orthodontic treatment history was not associated with OHRQoL in our sample. While the OHIP-14 is among the most commonly used tools for assessing OHRQoL, it has a functional bias and may lack sensitivity in capturing OHRQoL issues specifically related to orthodontic concerns [38]. A longitudinal study setting could have provided more insight into the subjective satisfaction and OHRQoL.

## Conclusions

In our cohort, the level of subjective orthodontic treatment need was low and OHRQoL was generally high among the 17-year-olds. The goal of orthodontic treatment in public health care, which is to achieve functional and esthetically acceptable occlusion, was achieved as reflected by the observed high level of satisfaction with dental esthetics and occlusal function in a group level. Nevertheless, the dissatisfaction of individual adolescents with dental esthetics and occlusal function was reflected as a lowered OHRQoL. Subjective assessment of treatment need correlated positively not only with a high OHIP score but also with a high PAR score. Therefore, correction of malocclusion could be anticipated to improve not only dental health but also subjective satisfaction with occlusion for individual adolescents.

## Data Availability

All data supporting the findings of this study are available within the paper and the supplementary online material. The original data are not openly available due to reasons of patient confidentiality.
